# Evaluation of DNA Extraction Methods for Reliable Quantification of *Acinetobacter baumannii*, *Klebsiella pneumoniae*, and *Pseudomonas aeruginosa*

**DOI:** 10.3390/bios13040463

**Published:** 2023-04-06

**Authors:** Alexandra Bogožalec Košir, Dane Lužnik, Viktorija Tomič, Mojca Milavec

**Affiliations:** 1 Department of Biotechnology and Systems Biology, National Institute of Biology, Večna Pot 111, 1000 Ljubljana, Slovenia; 2 University Clinic of Respiratory and Allergic Diseases Golnik, Golnik 36, 4204 Golnik, Slovenia

**Keywords:** nucleic acid, dPCR, DNA extraction methods, Gram-negative bacteria

## Abstract

Detection and quantification of DNA biomarkers relies heavily on the yield and quality of DNA obtained by extraction from different matrices. Although a large number of studies have compared the yields of different extraction methods, the repeatability and intermediate precision of these methods have been largely overlooked. In the present study, five extraction methods were evaluated, using digital PCR, to determine their efficiency in extracting DNA from three different Gram-negative bacteria in sputum samples. The performance of two automated methods (GXT NA and QuickPick genomic DNA extraction kit, using Arrow and KingFisher Duo automated systems, respectively), two manual kit-based methods (QIAamp DNA mini kit; DNeasy UltraClean microbial kit), and one manual non-kit method (CTAB), was assessed. While GXT NA extraction kit and the CTAB method have the highest DNA yield, they did not meet the strict criteria for repeatability, intermediate precision, and measurement uncertainty for all three studied bacteria. However, due to limited clinical samples, a compromise is necessary, and the GXT NA extraction kit was found to be the method of choice. The study also showed that dPCR allowed for accurate determination of extraction method repeatability, which can help standardize molecular diagnostic approaches. Additionally, the determination of absolute copy numbers facilitated the calculation of measurement uncertainty, which was found to be influenced by the DNA extraction method used.

## 1. Introduction

Molecular diagnostics have become increasingly used in pathogenic agent identification and detection based on specific biomarkers. One of the most commonly used molecular diagnostic methods is quantitative real-time polymerase chain reaction (qPCR). In qPCR, DNA biosensors in the form of a fluorescently labelled probe are used to detect a DNA biomarker. However, while molecular approaches are faster than traditional culture-based methods, they also experience measurement challenges. Factors, such as the efficiency of the measuring system and extraction method, sample variability, and the presence of inhibitors in samples, can all affect the accuracy of measurements [1].

DNA extraction is one of the first steps in these molecular diagnostic procedures, and the detection and quantification of specific biomarkers are highly dependent on the yield and quality of the extracted nucleic acids. A variety of different extraction kits for manual DNA extraction have been complemented by a wide range of different automated systems that have almost completely replaced ‘open’ extraction methods, such as cetyltrimethylammonium bromide (CTAB)-based extraction. However, it is important to note that DNA extraction cannot distinguish between the target biomarker and background DNA. As a result, accurately quantifying the target molecule is just as important as achieving a high yield. It is also important to consider not only the target DNA (e.g., virus, bacteria, plants, fungi, animals, etc.) and the matrix or background in which it is present, but also the downstream molecular method, as DNA fragmentation can significantly affect detection of the target. Additionally, cost and time efficiency are also important considerations in selecting a DNA extraction method.

A considerable number of comparative studies have been performed, focusing on the yield of two to three (and rarely more) extraction methods to determine the optimal method for extracting nucleic acids from a given pathogen. Manual and automated approaches have been studied to determine the most efficient methods for viral nucleic acid yields for both RNA viruses (e.g., noroviruses, HIV, and SARS-CoV-2) and DNA viruses (e.g., cytomegalovirus, hepatitis B) [2,3,4,5,6,7,8,9], as well as for bacterial DNA extraction from complex samples (e.g., faeces, blood, urine, sputum, and amniotic fluid) [10,11,12,13,14,15,16,17,18,19,20,21,22,23]. These comparisons are primarily based on differences in the number of qPCR cycles used, although there have also been studies in which comparisons have been based on spectrophotometry or fluorimetric detection [24,25] or PCR [26]. Although digital PCR (dPCR) has greater potential for absolute quantification and lower sensitivity to inhibitors, it has surprisingly rarely been used for comparisons of extraction methods. One exception was a large study, comparing 11 different extraction methods to determine the effect of DNA size on yield [27].

The objective of this study was to compare the efficacy of different DNA extraction methods for extracting DNA, including specific DNA biomarkers, from sputum samples containing three Gram-negative bacterial species: *Acinetobacter baumannii*, *Klebsiella pneumoniae*, and *Pseudomonas aeruginosa*. Three samples, labeled A, B, and C, were prepared, each containing all three bacterial species in varying concentrations. We tested five extraction methods, including two automated methods, using the GXT NA extraction kit on an Arrow system and QuickPick genomic (g) DNA extraction kit on a KingFisher Duo system; two manual kit-based methods used the QIAamp DNA mini kit and the DNeasy UltraClean microbial kit; and an ‘open’ CTAB method was used. The yield of each extraction method was quantified using digital PCR (dPCR), which allowed us to determine the absolute concentrations of DNA biomarkers specific to each bacterial species in copies per mL (cp/mL), instead of quantification cycle (Cq) values. This approach facilitated the accurate determination of the repeatability of each DNA extraction method and helped towards standardizing molecular diagnostic approaches. Furthermore, determination of the absolute copy numbers of DNA biomarkers facilitated the calculation of measurement uncertainty (MU), which revealed that MU was indeed influenced by the DNA extraction methods [28,29].

## 2. Material and Methods

The “Minimum Information for Publication of Quantitative Digital PCR Experiments for 2020” (dMIQE2020) checklist [30] was followed, and it is given in Supplementary Material.

### 2.1. Sample Preparation

The *A. baumannii* (30007), *K. pneumoniae* (30104), and *P. aeruginosa* (50071)-type strains were obtained from the German Collection of Microorganisms and Cell Cultures (DSMZ). Three suspensions that each contained one of these bacteria were prepared in sterile 30% glycerol in phosphate-buffered saline and adjusted to 1 × 10^8^ cells/mL based on turbidity measurements (DEN-1B densitometer; BioSan). These bacterial suspensions were subsequently diluted in 30% glycerol in phosphate-buffered saline as a series of 10-fold dilutions. The dilutions with 1 × 10^7^ cells/mL, 1 × 10^6^ cells/mL, and 1 × 10^5^ cells/mL of the individual bacterial species were used to prepare three samples (A, B, C), each of which contained all three of these bacterial species spiked in sputum in an asymmetric fashion; i.e., each bacterial species was present at a different concentration, as 5.5 × 10^5^ cells/mL, 5.5 × 10^4^ cells/mL, or 5.5 × 10^3^ cells/mL (Supplementary Table S1). These concentrations are classified as high, medium, and low in the manuscript. The samples were aliquoted into DNA low-binding tubes (200 µL per tube). All of these aliquots were then stored at <−70 °C until extraction.

Sputum, consisting of 16 units collected at the University Clinic for Respiratory and Allergic Diseases in Golnik (Slovenia), was initially tested for *A. baumannii*, *K. pneumonia*, and *P. aeruginosa*. The collected sputum was culture negative for these bacteria, while 12 units contained normal mixed respiratory flora, and four contained normal mixed respiratory flora and *S. aureus* (two samples), *H. influenzae* (one sample), and *S. pneumonia* (one sample). The sputum unites were subsequently pooled and digested by addition of Liquillizer (1:1; MetaSystems), a mucolytic agent used for liquefying respiratory samples.

In addition to the turbidity measurements, the concentration of each of the individual bacterial suspensions was evaluated using direct dPCR (Supplementary Table S2). Here, the suspensions used for the spiking (dilutions at 1 × 10^7^ cells/mL, 1 × 10^6^ cells/mL, 1 × 10^5^ cells/mL) were submitted to dPCR without prior DNA extraction (see Section 2.9 Digital PCR). Based on the results from this direct dPCR, the compositions of samples A–C were corrected (Supplementary Table S1).

### 2.2. Extraction Methods

**Figure 1:**
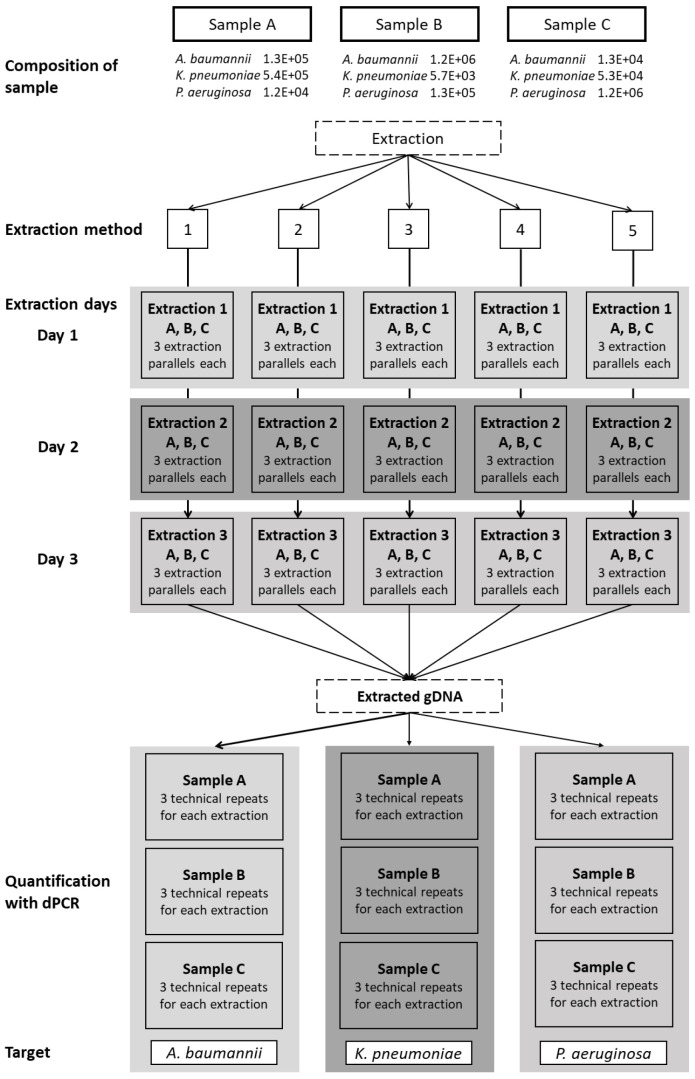
Schematic representation of the workflow. Three sputum samples, labeled A, B, and C, containing all three bacterial species in varying concentrations, were extracted using five extraction methods: 1, CTAB; 2, QIAamp DNA mini kit; 3, GXT NA extraction kit; 4, QuickPick genomic DNA extraction kit; 5, DNeasy UltraClean microbial kit. Each extraction was repeated on three different days, with three parallel extractions each day (three aliquots tested). The extracted genomic DNA was quantified using dPCR as three technical repeats for each of the target bacteria.

Each of the five extraction methods was repeated on three different days, with three aliquots per sample extracted on each day. For every extraction method, nine aliquots of each sample were extracted in total (Figure 1). A negative extraction control (200 µL water) was added to each extraction. All of the extracted DNA samples were stored in DNA low-binding tubes at <−20 °C.

### 2.3. The CTAB Method

The CTAB method was adapted based on Devonshire et al. [29] and was performed as follows: the thawed aliquots were briefly centrifuged, and 10 µL 50 mg/mL lysozyme was added to each. The samples were mixed by inverting the tubes 10 times, which was followed by an overnight incubation at 37 °C. The next day, the tubes were removed from the water bath, and 70 µL 10% sodium dodecyl sulphate and 5 µL 20 mg/mL proteinase K were added. The samples were mixed by inverting the tubes, and then they were incubated at 65 °C for 10 min. After this incubation, 100 µL, 5 M NaCl, and 100 µL pre-warmed 10% (*w*/*v*) CTAB/700 nM NaCl buffer were added, with the samples mixed by inverting the tubes, and they were incubated at 65 °C for 10 min. The samples were subsequently transferred to a fume hood where 750 µL chloroform/isoamyl alcohol (24:1, *v*/*v*) (Sigma-Aldrich, St. Louis, MO, USA) was added. The samples were mixed by inverting the tubes, followed by centrifugation at 10,000× *g* for 5 min. The upper aqueous supernatants were transferred to new tubes with 450 µL ice-cold isopropanol (Sigma- Aldrich, St. Louis, MO, USA), and they were chilled for 30 min at −20 °C. The samples were then centrifuged at 10,000× *g* for 15 min. The supernatants were removed and discarded, and the pellet was washed with 1 mL ice-cold 70% ethanol (Sigma- Aldrich, St. Louis, MO, USA). The tubes were centrifuged at 10,000× *g* for 5 min, and the ethanol was removed and discarded. The pellets were dried by incubation of the opened tubes at 65 °C until the ethanol had evaporated. The pellets were rehydrated in 200 µL TE buffer and left overnight at 4 °C to ensure complete re-suspension.

### 2.4. GXT NA Extraction Kit

The GXT NA extraction kit (Hain Lifescience, Nehren, Germany) was used in combination with an Arrow extraction system (NorDiag, Oslo, Norway). The thawed sample aliquots were diluted to 550 µL with phosphate-buffered saline, and then 10 µL proteinase K was added to each. The samples were incubated at 56 °C for 10 min. After this incubation, the samples were transferred to the Arrow instrument for extraction, and a protocol for extraction from 550 µL and elution in 100 µL was selected. The protocol consists of lysis, coupling with magnetic beads, washing, and elution.

### 2.5. QuickPick Genomic DNA Extraction Kit

The QuickPick gDNA extraction kit (BioNobile, Pargas; Finland) was used in combination with a KingFisher Duo extraction system (ThermoFisher Scientific, Waltham, MA, USA). The samples were thawed and centrifuged at 10,000× *g* for 1 min, the supernatants were removed, and the samples were centrifuged for an additional 30 s at 10,000× *g*. Again, the supernatants were removed, and the pellets were resuspended in 100 µL lysis buffer and 20 µL proteinase K. The samples were mixed by vortexing, and then, they were incubated at 56 °C for 30 min. Each deep-well 96-well plate was filled with 8 µL MagaZorb magnetic particles and 250 µL binding buffer (row 1), 500 µL wash buffer 1 (rows 3, 4), and 500 µL wash buffer 2 (row 5). The samples were eluted in 50 µL elution buffer. The extraction protocol selected was for Quick Pick gDNA tissue (provided by the kit manufacturer).

### 2.6. DNeasy Ultraclean Microbial Kit

The samples were thawed and centrifuged at 10,000× *g* for 1 min. The supernatants were removed and discarded, and the tubes were centrifuged again at 10,000× *g* for 30 s. The supernatants were removed. The pellets were resuspended in 300 µL power-bead solution (as provided), and the DNA was extracted according to the DNeasy Ultraclean microbial kit (Qiagen, Hilden, Germany) manual, with elution in 50 µL elution buffer.

### 2.7. QIAamp DNA Mini Kit

The samples were thawed and centrifuged at 10,000× *g* for 1 min. The supernatants were removed and discarded, which was followed by another centrifugation step at 10,000× *g* for 30 s. The supernatants were removed, and the pellets were resuspended in 180 µL ATL buffer (as supplied). Then, 20 µL proteinase K was added, and the tubes were incubated at 56 °C for 1 h. After this incubation, 200 µL AL buffer (as supplied) was added, and the tubes were incubated at 70 °C for 10 min. Then, 200 µL ethanol (96–100%) was added, and the samples were mixed by pulse vortexing for 15 s, and then they were transferred to the QIAamp mini spin columns. After 1 min of centrifugation at 6000× *g*, the spin columns were transferred to clean tubes, and 500 µL AW1 buffer (as supplied) was added. The columns were centrifuged at 6000× *g* for 1 min. The collection tubes were discarded, and the spin columns were placed in clean tubes. Then, 500 µL AW2 buffer (as supplied) was added, and the columns were centrifuged at 20,000× *g* for 3 min. The collection tubes were discarded, and the spin columns were centrifuged again at 20,000× *g* for 1 min. The spin columns were transferred to clean tubes, and 100 µL AE buffer (as supplied) was added to each, with the spin columns then incubated at room temperature for 5 min. The DNA was eluted by centrifuging the spin columns at 6000× *g* for 1 min.

### 2.8. DNA Quality Assessment

The integrity of the extracted DNA was assessed using gel electrophoresis, with either a 2100 Bioanalyzer (Agilent Technologies, Santa Clara, CA, USA) or a LabChip GX (PerkinElmer, Waltham, MA, USA) instrument. On the first day of extraction, all three of the parallel extractions for all three of the samples (A–C) were tested using each of the extraction methods (Supplementary Figures S1 and S2). Additionally, a spectrophotometer (NanoDrop 1000; ThermoFisher Scientific) was used to determine the purity of the extracted DNA. On the first day of extraction, we tested the DNA from one parallel extraction of sample A by using 1 μL of the extracted DNA (see Supplementary Table S3).

### 2.9. Digital PCR

The dPCR reactions (total volume, 20 μL) contained 10 μL ddPCR Supermix for probes (no dUTP; Cat. No. 1863024; BioRad, Pleasanton, CA, USA), 6 μL primers and probe mix, and 4 μL DNA. All of the methods were developed in-house (Supplementary Table S4, paper on validation of methods in preparation). For the droplet generation, droplet generator cartridges (DG8; BioRad) were combined with the droplet digital system (QX100/QX200; BioRad). The droplets generated were transferred to 96-well plates, and the PCR reactions were carried out using a thermal cycler (C1000 or T100; BioRad, USA) under the following amplification conditions: 10 min DNA polymerase activation at 95 °C; followed by 40 cycles of a two-step thermal profile of 30 s at 94 °C for denaturation and 60 s at 60 °C for annealing and extension; this was followed by 10 min at 98 °C; and then, cooling to 4 °C was performed. After the thermal cycling, the 96-well plates were transferred to a droplet reader (QX100/QX200; BioRad), and the data were gathered.

The data were analysed using the software package provided with the dPCR system (QuantaSoft 1.7.4.0917; BioRad) and Microsoft Excel. The rejection criterion was set to exclude reactions from subsequent analysis when/if the number of accepted droplets was <8000 per 20 μL PCR. Each sample was tested in triplicate, and each negative control of extraction was tested in duplicate. Additionally, direct dPCR of each suspension used for the spiking was performed under the same conditions. Each suspension was tested in quadruplicate. Each dPCR run contained a negative-template control and a positive control for each of the assays. The concentration of the input DNA was calculated in copies per µL based on Equation (1),

(1) $$c=\frac{\ln\left(1-\frac{N_{pos}}{N_{all}}\right)}{\left(\frac{V_{droplet}}{V_{reaction}}\right)\times V_{added\;DNA}}$$

where, *N_pos_* is the number of positive droplets, *N_all_* is the number of all of the accepted droplets, *V_droplet_* is the droplet volume based on Bogožalec Košir et al. [31], *V_reaction_* is the reaction volume (20 µL), and *V_added DNA_* is the volume of the DNA sample in the reaction (4 µL).

The yields for each of the extraction methods were calculated in copies per mL based on Equation (2):

(2) $$yield=\frac{c\times V_{elution}}{V_{sample}}\times 1000$$

where *c* is the concentration in copies per µL (Equation (1)), *V_sample_* is the volume of sample (200 µL for each of the extraction methods), and *V_elution_* is the elution volume, which changed according to the method used: 50 µL for DNeasy UltraClean microbial kit and QuickPick gDNA extraction kit on the KingFisher Duo instrument, 100 µL for QIAamp DNA mini kit and GXT NA extraction kit on the Arrow instrument, and 200 µL for the CTAB method.

### 2.10. Statistical Analysis

To numerically determine the efficiency of each extraction method for the extraction of the bacterial DNA from these complex samples, a bias (%) of the yield to the theoretical 100% yield was calculated using Equation (3).

(3) $$bias=\frac{\left(yield-yield\;theoretical\right)}{yield\;theoretical}\times 100$$

To determine the repeatability and intermediate precision of each extraction method, the coefficients of variation (CVs; %,) were calculated using Equation (4). Repeatability was determined for each extraction method for each individual day (nine measurements, for three parallel extractions), while intermediate precision was determined as the variability between the mean concentration (cp/mL) obtained for each of the three days of extraction (three measurements, one for each day of extraction). For both the repeatability and the intermediate precision, the acceptability criteria were set at CV ≤ 25%.

(4) $$CV=\frac{SD_{measurement}}{mean_{measurement}}\times 100$$

The bottom-up approach was used to determine the expanded MU for each DNA extraction method and each sample [32]. A combined measurement uncertainty, *u_c_*, was calculated according to Equation (4),

(5) $$u_{c}=\sqrt{u_{r}{}^{2}+u_{ip}{}^{2}+u_{v}{}^{2}\;}$$

where *u_r_* is the uncertainty associated with repeatability, *u_ip_* is the uncertainty associated with intermediate precision, and *u_r_* is the partition volume uncertainty based on Bogožalec Košir et al. [31]. *u_r_* and *u_ip_* (*u_ip_*_1_ or *u_ip_*_2_) were calculated according to Equations (6)–(8):

(6) $$u_{r}=\frac{\sqrt{MS_{\mathrm{within}}}\;}{\sqrt{n}}\;$$

(7) $$u_{ip1}=\sqrt{\frac{MS_{\mathrm{between}}-MS_{\mathrm{within}}}{n\times N}}$$

(8) $$\;\;\;\;\;\;\;\;\;\;\;\;\;\;\;\;\;\;\;\;\;\;\;\;\;u_{ip2}=\frac{\sqrt{\frac{MS_{\mathrm{within}}\;}{n}}\times \sqrt[{4}]{\frac{2}{N\times \left(n-1\right)}}}{\sqrt{N}}$$

where *n* is the number of independent replicates per experiment i.e., the number of technical repeats performed in dPCR per individual extraction parallel, *N* is the number of experiments performed, i.e., the number of extraction parallels or sample aliquots tested, *MS*_within_ is the mean square value within groups, i.e., within experiment, and *MS*_between_ is the mean square value between groups, i.e., between experiments. Both of these mean squares were calculated using ANOVA in Microsoft Excel 2016, with all of the measurements taken into account. If *MS*_between_ > *MS*_within_, Equation (7) was used to calculate the uncertainty associated with intermediate precision. Otherwise, Equation (8) was used. A coverage factor (*k*) of 2.1 was chosen at the 95% level of confidence based on the degrees of freedom, and it was applied to obtain the expanded MU. MU is expressed as a relative value (%).

## 3. Results and Discussion

Testing DNA extraction methods on complex samples is critical in molecular diagnostics, even though extracting DNA from a complex sample can be challenging, particularly when detecting low concentration biomarkers in a high background. Samples, such as sputum, blood, or urine, can contain not only bacteria that cause the infection, but also dead cells, cell debris, white blood cells, and other immune cells, where bacterial DNA accounts for only a small fraction of the total extracted DNA. With such complexity, there is a possibility that all components may not be extracted with the same efficiency, and the remaining impurities could affect downstream analysis. To contribute to standardization in molecular diagnostics, only well defined extraction methods should be used. Thus, this study aimed to test the efficiencies of different DNA extraction methods to extract DNA from Gram-negative bacteria in a complex sample, such as sputum, as well as the repeatability of these methods (Figure 1). The method of choice was dPCR due to its higher accuracy and precision and tolerance to PCR inhibitors.

To encompass the various extraction approaches in use today, five extraction methods were selected, each with its own characteristics. One of the most widely used open methods is one based on CTAB. However, CTAB is not just one method; many different CTAB-based extraction protocols have been developed for various applications, including the extraction of bacterial DNA from sputum samples [29]. CTAB-based methods are more cost-effective; however, the trade-off is speed. The much faster column-based extraction methods have also been used, although they are more expensive compared to the open methods. Based on a search of the literature, the QIAamp DNA mini kit and the DNeasy Ultra-Clean microbial kit were selected. The fastest methods for DNA extraction are the autocombined methods. There is a plethora of kits on the market that are compatible with one or more nucleic acid extraction robots. In the present study, the following combinations were chosen: GXT NA Extraction Kit on an Arrow system and QuickPick gDNA Extraction Kit on a KingFisher Duo system. The first combination is used in routine diagnostics at the University Clinic of Respiratory Diseases and Allergies in Golnik (Slovenia), while the KingFisher extraction robots are used in combination with different nucleic acid extraction kits for both research and pathogen diagnostics at the National Institute of Biology in Ljubljana (Slovenia).

Bacterial concentrations used in the spiking solutions were initially evaluated by turbidity measurements (McFarland units), which were later corrected by direct dPCR (Supplementary Table S1). Amplification was performed without prior extraction of bacterial DNA (see Supplementary Figure S3 for amplification profile), which has been shown to be an accurate quantification approach for both bacteria and viruses [33,34]. Because each of the three dPCR methods targets a specific sequence in one copy in the bacterial genome, data from dPCR (cp/mL; i.e., copy number concentration) are comparable to bacterial concentrations in cells/mL. In this way, the data obtained with turbidimetry and direct dPCR can be compared, and any variations between the methods can be taken into account when determining the theoretical 100% extraction yield. This comparison showed that the turbidimetry and direct dPCR methods gave similar concentrations for *K. pneumoniae*, whereas the concentrations estimated by direct dPCR were twice that of the turbidimetry for both *A. baumannii* and *P. aeruginosa* (Supplementary Table S1). As different bacterial species differ in size and mass, turbidity measurements are more reliable for species similar to *E. coli*, for which the standards were established [35]. Therefore, in the present study, the theoretical 100% yield of bacterial DNA based on direct dPCR was corrected to the following concentrations: *A. baumannii*: low, ~1.3 × 10^4^ cp/mL; medium, ~1.3 × 10^5^ cp/mL; high, ~1.2 × 10^6^ cp/mL; *K. pneumoniae*: low, ~5.7 × 10^3^ cp/mL; medium, ~5.3 × 10^4^ cp/mL; high, ~5.4 × 10^5^ cp/mL; and *P. aeruginosa*: low, ~1.2 × 10^4^ cp/mL; medium, ~1.3 × 10^5^ cp/mL; high, ~1.2 × 10^6^ cp/mL (Supplementary Table S1).

### 3.1. Evaluation of DNA Quality

Based on spectrophotometric evaluation, DNA can be considered pure when the absorbance ratio of A260/280 is ~1.8 and the secondary absorbance ratio of A260/230 is 1.8 to 2.2. Here, the A260/280 ratio was ~1.8 for all five extraction methods, whereas the A260/230 ratio was ~2.0 for the QIAamp DNA mini kit and the QuickPick gDNA extraction kit, 2.30 for the DNeasy UltraClean microbial kit, 1.44 for the CTAB method, and 1.33 for the GXT NA extraction kit (Supplementary Table S3). Although spectrophotometric measurements give an indication of DNA purity, a ratio outside the optimal range does not necessarily indicate reduced amplification, as has been shown previously even for samples as complex as vegetable oil [36]. Based on the amount of “rain”, i.e., positive partitions between the negative and positive cluster, and the amplitude of fluorescence of the positive clusters in dPCR analysis, which did not differ between extraction methods, the suboptimal A260/280 ratio did not affect DNA amplifiability.

Fragmentation is also a factor that can affect DNA amplification. The data from electrophoresis showed two distinct fragmentation patterns, with the QIAamp DNA mini kit, QuickPick gDNA extraction kit, and UltraClean Microbial kit have longer fragments between 1 kbp and 10 kbp. In contrast, the CTAB method and the GXT NA extraction kit yielded shorter fragments between 50 bp and 5 kbp, with a peak of ~200 bp (Supplementary Figures S1 and S2). In qPCR and dPCR methods, and especially in methods based on fluorescent probes, the optimal length of amplicons to promote efficient amplification is between 60 bp and 90 bp [37]. Therefore, even with their more fragmented DNA, the CTAB method and the GXT NA extraction kit were still fit for their purpose.

### 3.2. Evaluation of Extraction Yields

**Figure 2:**
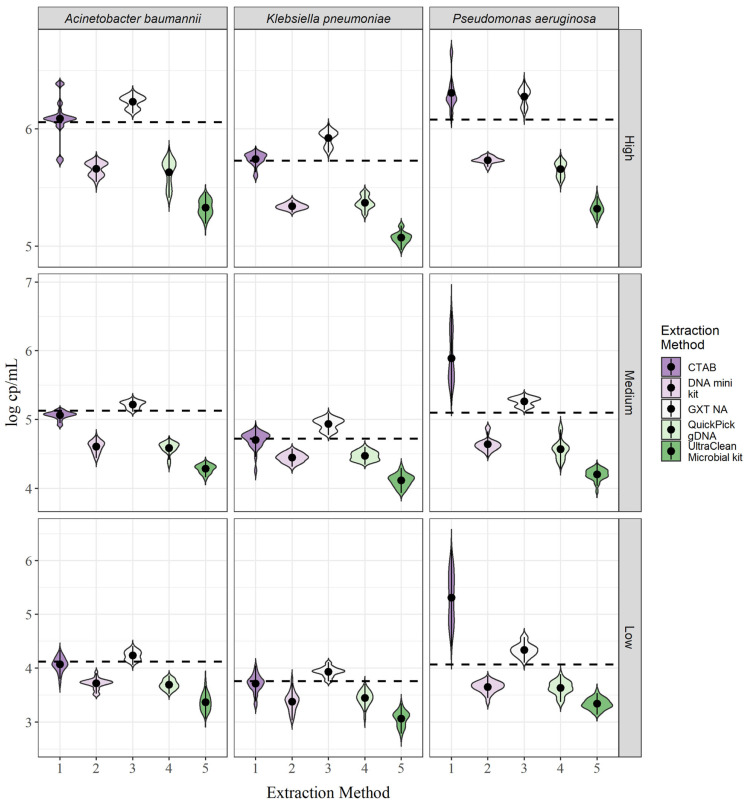
Yields of DNA biomarkers for each of the target bacteria and DNA extraction methods. The dotted line represents the theoretical 100% yield.

For all three Gramme-negative bacteria, large differences in targeted DNA yields were observed between the different extraction methods (Figure 2). Although the differences in yield varied between bacterial species, a trend in extraction efficiency was observed. For all three bacterial species, yields were highest with the GXT NA extraction kit and the CTAB method, followed by the QIAamp DNA mini kit, the QuickPick gDNA extraction kit, and the DNeasy UltraClean microbial kit (Figure 2). The GXT NA extraction kit showed a slightly higher yield than the CTAB method for *A. baumannii* and *K. pneumoniae*, and the reverse was true with CTAB for *P. aeruginosa*. In addition, the trends were the same for all three bacterial concentrations. In all cases, the yields exceeded the theoretical 100% yield thresholds (Figure 2, dashed lines) for the GXT NA extraction kit, whereas, for the CTAB method, the threshold was exceeded only for *P. aeruginosa*, and for *A. baumannii* and *K. pneumoniae*, the mean concentrations were within a 15% bias (Table 1; Supplementary Figure S4). For a numerical comparison of yields, the bias (see Equation (3)) to the theoretical 100% yield was calculated for each of the extraction methods and each of the bacterial concentrations (Table 1). In general, the concentrations did not affect the bias; for example, the bias for *K. pneumoniae* using the QIAamp DNA mini kit was −69.4%, −60.0%, and −60.0% for the low, medium, and high concentrations, respectively (Table 1; Supplementary Figure S4). The variation was approximately in the same range for all bacterial species. The exception was the CTAB method for *A. baumannii* and *P. aeruginosa*, with a large variability between concentrations for *A. baumannii* and a much larger bias for *P. aeruginosa* compared with the other two bacterial species (Supplementary Figure S4).

### 3.3. Repeatability and Intermediate Precision

To compare these methods, it is not sufficient to determine only which extraction method gives the highest yield of biomarkers, but it is also necessary to evaluate which method is the most reliable in terms of repeatability and intermediate precision.

Repeatability was calculated separately for each of the concentrations for each of the bacterial species. For *A. baumannii*, none of the extraction methods consistently had the lowest CV (Supplementary Table S5). For *K. pneumoniae* and *P. aeruginosa*, the lowest CV was observed for the GXT NA extraction kit, except for the low concentration of *P. aeruginosa*, where none of the methods consistently had the lowest CV over three days (Supplementary Tables S6 and S7).

The criteria of CV ≤ 25% for repeatability was achieved for all five extraction methods only for the medium concentration of *A. baumannii* (4.1−19.8%) and for the high concentrations of *K. pneumoniae* (4.0–17.2%) and *P. aeruginosa* (3.3–17.9%). This was true with the exception of the CTAB method for *P. aeruginosa*, where, for day three, the CV was 42.0% (Supplementary Tables S5–S7). For *A. baumannii*, only the GXT NA extraction kit and the DNeasy UltraClean microbial kit had a CV of ≤25% for all three days and all three concentrations (Supplementary Table S5). The same was true for *P. aeruginosa* for the high and medium concentrations, whereas, for the low concentration of *P. aeruginosa*, the CV was >25% for at least one of the three extraction days (Supplementary Table S7). Only the GXT NA -extraction kit had CVs < 25% for *K. pneumoniae* on all three days and for all three concentrations (Supplementary Table S6).

Repeatability was reflected in the shapes of the violin plots shown in Figure 2. For *A. baumannii*, a distinct peak was detected for all extraction methods for the medium concentration compared to the low and high concentrations, where two peaks were generally more common (Figure 2). For the high concentration, the CTAB method even showed two peaks and three outliers in the violin plot, indicating high variability between parallel extractions. For *K. pneumoniae*, two peaks were observed in some cases, with the most striking example being the GXT NA extraction kit for the medium and high concentrations and the QuickPick gDNA extraction kit (Figure 2). This effect was observed to a lesser extent in *P. aeruginosa*. Measurements varied for *P. aeruginosa*, especially for the CTAB method, where no clear peak was seen (Figure 2). This suggests that the lysis step is less efficient for *P. aeruginosa* compared to the other two bacteria and would need to be optimized if the method is to be used routinely.

**Figure 3:**
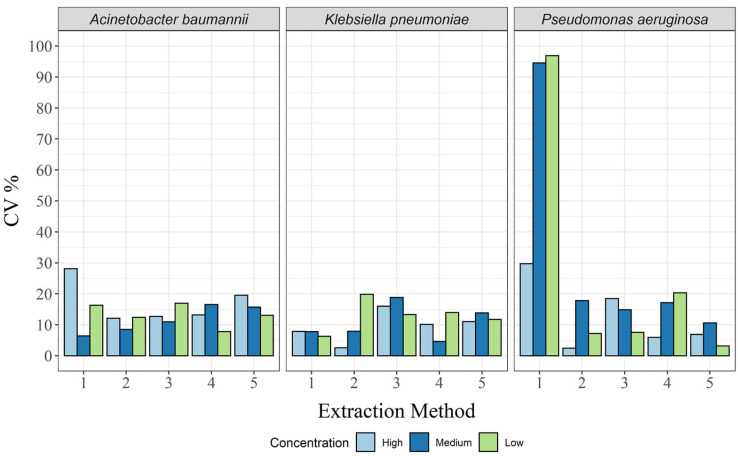
Intermediate precision in CV%, for each of the three bacterial species and for all extraction methods: 1, CTAB; 2, QIAamp DNA mini kit; 3, GXT NA extraction kit; 4, QuickPick genomic DNA extraction kit; 5, DNeasy UltraClean microbial kit.

Compared with repeatability, the variability between days (intermediate precision) was low, with CVs < 20% in almost all cases (Figure 3 and Supplementary Tables S8–S10). The exception here was the CTAB method, where the CV was 28.1% for *A. baumannii* at the high concentration, as well as 96.9%, 94.6%, and 29.7% for *P. aeruginosa* at the low, medium, and high concentrations, respectively (Supplementary Tables S8 and S10). Thus, all extraction methods, except the CTAB method, were within the acceptance criteria (CV < 25%) for all concentrations and all bacterial species.

### 3.4. Measurement Uncertainty

**Figure 4:**
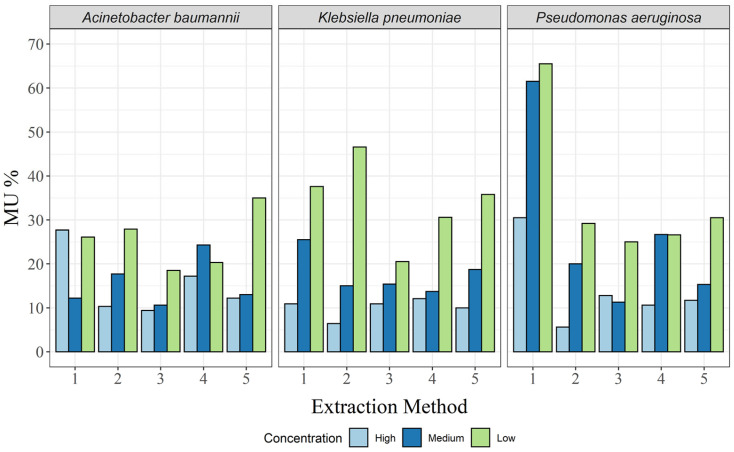
Relative expanded (k = 2) measurement uncertainty for each of the three bacterial species and for all extraction methods: 1, CTAB; 2, QIAamp DNA mini kit; 3, GXT NA extraction kit; 4, QuickPick genomic DNA extraction kit; 5, DNeasy UltraClean microbial kit.

No such analysis is complete without the associated MU, which was calculated here as a separate relative MU for each targeted bacterial DNA concentration. As expected, MU decreased with increasing target DNA concentration in almost all cases, except for the CTAB method and the QuickPick gDNA extraction kit for *A. baumannii* (Table 2; Figure 4). For the CTAB method, MU was highest at the high concentration, and for the QuickPick gDNA extraction kit, MU was highest at the medium concentration.

For *A. baumannii*, the GXT NA extraction kit had the lowest MU for all three concentrations, while the highest MUs were seen for the DNeasy UltraClean microbial kit, QuickPick gDNA extraction kit, and CTAB method for the low, medium, and high concentrations, respectively (Table 2). For *K. pneumoniae*, none of the methods clearly had the lowest MUs (Table 2). However, the GXT NA extraction kit had the lowest difference between the different bacterial concentrations (Figure 4). For *P. aeruginosa*, very high MUs were observed for the CTAB method for the high and medium concentrations (65.5% and 61.5%, respectively), and although there was a significant decrease in MU at the low concentration (30.5%), it was still more than twice that of the other extraction methods at the same concentration (Table 2; Figure 4).

## 4. Conclusions

Based on the data obtained in this study, it is difficult to define an extraction method that would be best for all three of these bacterial species at all three concentrations tested. The overall quality of the extracted DNA was fit for purpose for all extraction methods, although it was more fragmented with the CTAB method and the GXT NA extraction kit. If we focus on biomarker yield as the main criterion, the GXT NA extraction kit and the CTAB method would be the first choices. However, the chosen extraction method must provide repeatable results every time, especially if it is to be used as a reference method or to characterize reference materials.

According to our criteria for repeatability and intermediate precision of these methods (MU ≤ 25%), none of these methods met the criteria for all three bacterial species. However, since clinical samples cannot be obtained in such quantities that the DNA of each organism can be extracted by a different method, a compromise needs to be reached here. According to these data, combining the yield and repeatability tests indicates that the GXT NA extraction kit would be the method of choice, with the QIAamp DNA mini kit is the second choice, especially for laboratories where automation of the extraction is not cost effective.

## Figures and Tables

**Table 1:** Bias to the theoretical 100% yield (based on direct dPCR) across the extraction systems for all three target bacteria.

Sample	Extraction Method	*A. baumannii*		*K. pneumoniae*		*P. aeruginosa*	
		(cp/mL)	Bias (%)	(cp/mL)	Bias (%)	(cp/mL)	Bias (%)
A	CTAB	1.2 × 10^5^	−13.0	5.6 × 10^5^	2.7	3.2 × 10^5^	2636.3
	QIAamp DNA mini kit	4.1 × 10^4^	−69.4	2.2 × 10^5^	−59.4	4.6 × 10^3^	−61.4
	GXT NA extraction kit	1.7 × 10^5^	24.1	8.5 × 10^5^	56.13	2.3 × 10^4^	89.72
	QuickPick genomic DNA kit	4.1 × 10^4^	−69.7	2.4 × 10^5^	−56.4	4.5 × 10^3^	−62.4
	DNeasy UltraClean microbial kit	1.9 × 10^4^	−85.5	1.2 × 10^5^	−78.1	2.2 × 10^3^	−81.2
B	CTAB	1.3 × 10^6^	13.3	5.5 × 10^3^	−3.9	1.1 × 10^6^	756.8
	QIAamp DNA mini kit	4.6 × 10^5^	−60.0	2.6 × 10^3^	−54.9	4.5 × 10^4^	−64.3
	GXT NA extraction kit	1.7 × 10^6^	48.8	8.7 × 10^3^	52.53	1.9 × 10^5^	48.28
	QuickPick genomic DNA kit	4.4 × 10^5^	−62.0	2.9 × 10^3^	−49.1	3.9 × 10^4^	−68.6
	DNeasy UltraClean microbial kit	2.2 × 10^5^	−81.4	1.2 × 10^3^	−78.6	1.6 × 10^4^	−87.1
C	CTAB	1.2 × 10^4^	−8.0	5.2 × 10^4^	−1.8	2.1 × 10^6^	79.3
	QIAamp DNA mini kit	5.3 × 10^3^	−60.0	2.8 × 10^4^	−46.9	5.4 × 10^5^	−54.7
	GXT NA extraction kit	1.7 × 10^4^	31.3	8.7 × 10^4^	63.96	1.9 × 10^6^	59.81
	QuickPick genomic DNA kit	5.0 × 10^3^	−62.4	3.0 × 10^4^	−43.5	4.6 × 10^5^	−61.8
	DNeasy UltraClean microbial kit	2.3 × 10^3^	−82.6	1.3 × 10^4^	−74.9	2.1 × 10^5^	−82.5

**Table 2:** Copy numbers and corresponding relative measurement uncertainties across the extraction systems for all three target bacteria.

Sample	Extraction Method	*A. baumannii*		*K. pneumoniae*		*P. aeruginosa*	
		(cp/mL)	MU (%)	(cp/mL)	MU (%)	(cp/mL)	MU (%)
A	CTAB	1.2 × 10^5^	12.2	5.6 × 10^5^	10.9	3.2 × 10^5^	65.5
	QIAamp DNA mini kit	4.1 × 10^4^	17.7	2.2 × 10^5^	6.4	4.6 × 10^3^	29.2
	GXT NA extraction kit	1.7 × 10^5^	10.6	8.5 × 10^5^	10.9	2.3 × 10^4^	25.0
	QuickPick genomic DNA kit	4.1 × 10^4^	24.3	2.4 × 10^5^	12.1	4.5 × 10^3^	26.6
	DNeasy UltraClean microbial kit	1.9 × 10^4^	13.0	1.2 × 10^5^	10.0	2.2 × 10^3^	30.5
B	CTAB	1.3 × 10^6^	27.7	5.5 × 10^3^	37.6	1.1 × 10^6^	61.5
	QIAamp DNA mini kit	4.6 × 10^5^	10.3	2.6 × 10^3^	46.6	4.5 × 10^4^	20.0
	GXT NA extraction kit	1.7 × 10^6^	9.4	8.7 × 10^3^	20.5	1.9 × 10^5^	11.3
	QuickPick genomic DNA kit	4.4 × 10^5^	17.2	2.9 × 10^3^	30.6	3.9 × 10^4^	26.7
	DNeasy UltraClean microbial kit	2.2 × 10^5^	12.2	1.2 × 10^3^	35.8	1.6 × 10^4^	15.3
C	CTAB	1.2 × 10^4^	26.1	5.2 × 10^4^	25.5	2.1 × 10^6^	30.5
	QIAamp DNA mini kit	5.3 × 10^3^	27.9	2.8 × 10^4^	15.0	5.4 × 10^5^	5.6
	GXT NA extraction kit	1.7 × 10^4^	18.5	8.7 × 10^4^	15.4	1.9 × 10^6^	12.8
	QuickPick genomic DNA kit	5.0 × 10^3^	20.3	3.0 × 10^4^	13.7	4.6 × 10^5^	10.6
	DNeasy UltraClean microbial kit	2.3 × 10^3^	35.0	1.3 × 10^4^	18.7	2.1 × 10^5^	11.7

## Data Availability

The data presented in this study are available in Supplementary Material, and no additional data were created or analyzed in this study.

## Supplementary Materials

The following supporting information can be downloaded at: https://www.mdpi.com/article/10.3390/bios13040463/s1, Figure S1: Integrity of the extracted DNA measured with the 2100 Bioanalyzer (Agilent) in combination with Agilent DNA 12,000 kits; Figure S2: Integrity of the extracted DNA measured with the LabChip GX capillary gel electrophoresis; Figure S3: Direct dPCR for the three bacterial solutions used for the spiking of the sputum samples; Figure S4: Bias to the theoretical 100% yields for the DNA extraction methods; Table S1: Compositions of samples A, B and C (spiked sputum samples with added Liquillizer) based on the turbidity measurements and corrected using direct dPCR analysis; Table S2: Concentrations and coefficients of variation (CV) of the spiking solutions, as measured by direct dPCR; Table S3: Purity of the extracted DNA, based on spectrophotometric measurem; Table S4: Primers and probes used in the dPCR quantification; Table S5: Repeatability with coefficient of variance (CV) for *A. baumannii* for the three bacterial concentrations; Table S6: Repeatability with coefficient of variance (CV) for *K. pneumoniae* for the three bacterial concentrations; Table S7: Repeatability with coefficient of variance (CV) for *P. aeruginosa* for the three bacterial concentrations; Table S8: Intermediate precision with coefficient of variance (CV) for *A. baumannii* for the three bacterial concentrations; Table S9: Intermediate precision with coefficient of variance (CV) for *K. pneumoniae* for the three bacterial concentrations; Table S10: Intermediate precision with coefficient of variance (CV) for *P. aeruginosa* for the three bacterial concentrations.
