# Supplementary material for: Evaluation of DNA Extraction Methods for Reliable Quantification of Acinetobacter baumannii, Klebsiella pneumoniae, and Pseudomonas aeruginosa
Source: Biosensors (Basel). 2023 Apr 6;13(4):463. doi: 10.3390/bios13040463 (PMC10136035; doi:10.3390/bios13040463)
Supplement: Supplementary file 1 [file biosensors-13-00463-s001.zip › biosensors-2251817-supplementary.pdf]

Supplementary Material To:

Evaluation of DNA Extraction Methods for Reliable Quantification of *Acinetobacter baumannii, Klebsiella pneumoniae* and *Pseudomonas aeruginosa*

Table of Contents

Supplementary Figures 2

Figure S1: Integrity of the extracted DNA measured with the 2100 Bioanalyzer (Agilent) in combination with Agilent DNA 12000 kits. 2

Figure S2: Integrity of the extracted DNA measured with the LabChip GX capillary gel electrophoresis. 3

Figure S3: Direct dPCR for the three bacterial solutions used for the spiking of the sputum samples. 4

Figure S4: Bias to the theoretical 100% yields for the DNA extraction methods 5

Supplementary Tables 6

Table S1: Compositions of samples A, B and C (spiked sputum samples with added Liquillizer) based on the turbidity measurements and corrected using direct dPCR analysis. .. 6

Table S2: Concentrations and coefficients of variation (CV) of the spiking solutions, as measured by direct dPCR. 7

Table S3: Purity of the extracted DNA, based on spectrophotometric measurements. 8

Table S4: Primers and probes used in the dPCR quantification. 9

Table S5: Repeatability with coefficient of variance (CV) for *A. baumannii* for the three bacterial concentrations. 10

Table S6: Repeatability with coefficient of variance (CV) for *K. pneumoniae* for the three bacterial concentrations. 11

Table S7: Repeatability with coefficient of variance (CV) for *P. aeruginosa* for the three bacterial concentrations. 12

Table S8: Intermediate precision with coefficient of variance (CV) for *A. baumannii* for the three bacterial concentrations. 13

Table S9: Intermediate precision with coefficient of variance (CV) for *K. pneumoniae* for the three bacterial concentrations. 14

Table S10: Intermediate precision with coefficient of variance (CV) for *P. aeruginosa* for the three bacterial concentrations. 15

Minimum Information for Publication of Quantitative Digital PCR Experiments for 2020" (dMIQE2020) checklist 16

Supplementary Figures

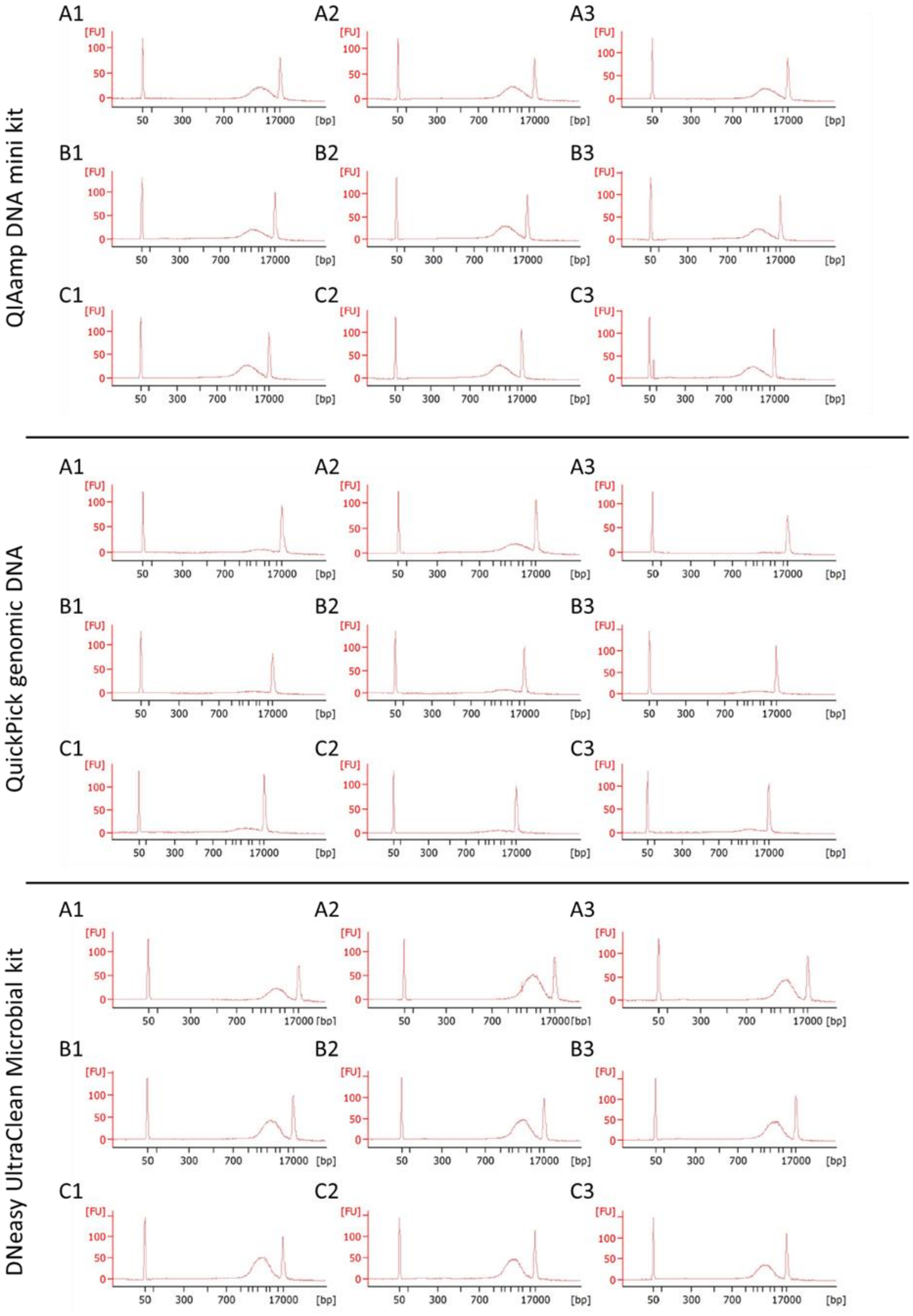

Figure S1: Integrity of the extracted DNA measured with the 2100 Bioanalyzer (Agilent) in combination with Agilent DNA 12000 kits. A1, A2, A3, all three extraction parallels of sample A, extracted on the first day of extraction; B1, B2, B3, all three extraction parallels of sample B, extracted on the first day of extraction; C1, C2, C3, all three extraction parallels of sample C, extracted on the first day of extraction; FU, arbitrary fluorescence units.

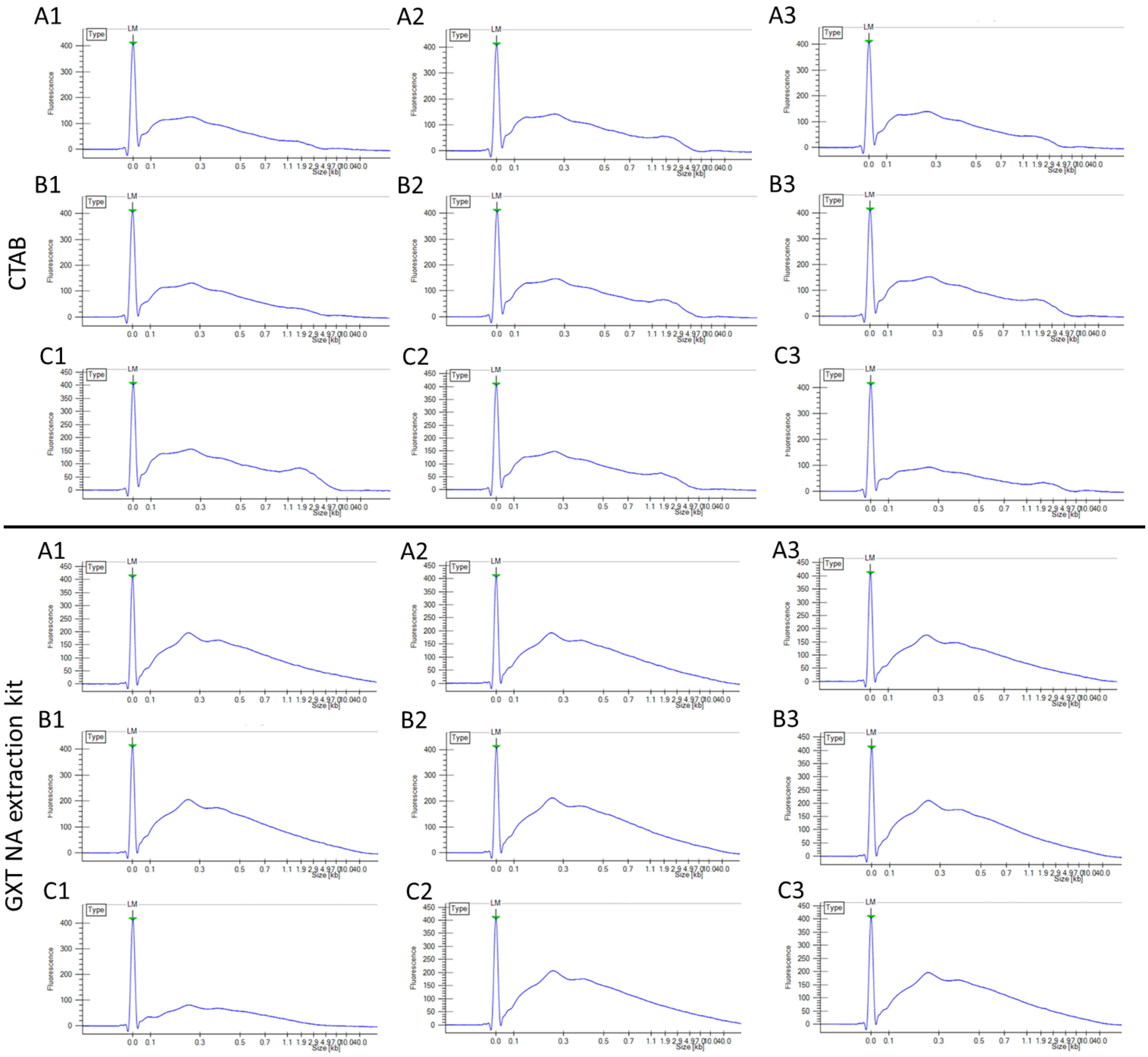

Figure S2: Integrity of the extracted DNA measured with the LabChip GX capillary gel electrophoresis. A1, A2, A3, all three extraction parallels of sample A, extracted on the first day of extraction; B1, B2, B3, all three extraction parallels of sample B, extracted on the first day of extraction; C1, C2, C3, all three extraction parallels of sample C; The ticks on the X axis represent the following lengths in bp: 0, 100, 300, 500, 700, 1100, 1900, 2900, 4900, 7000, 10000, 40000.

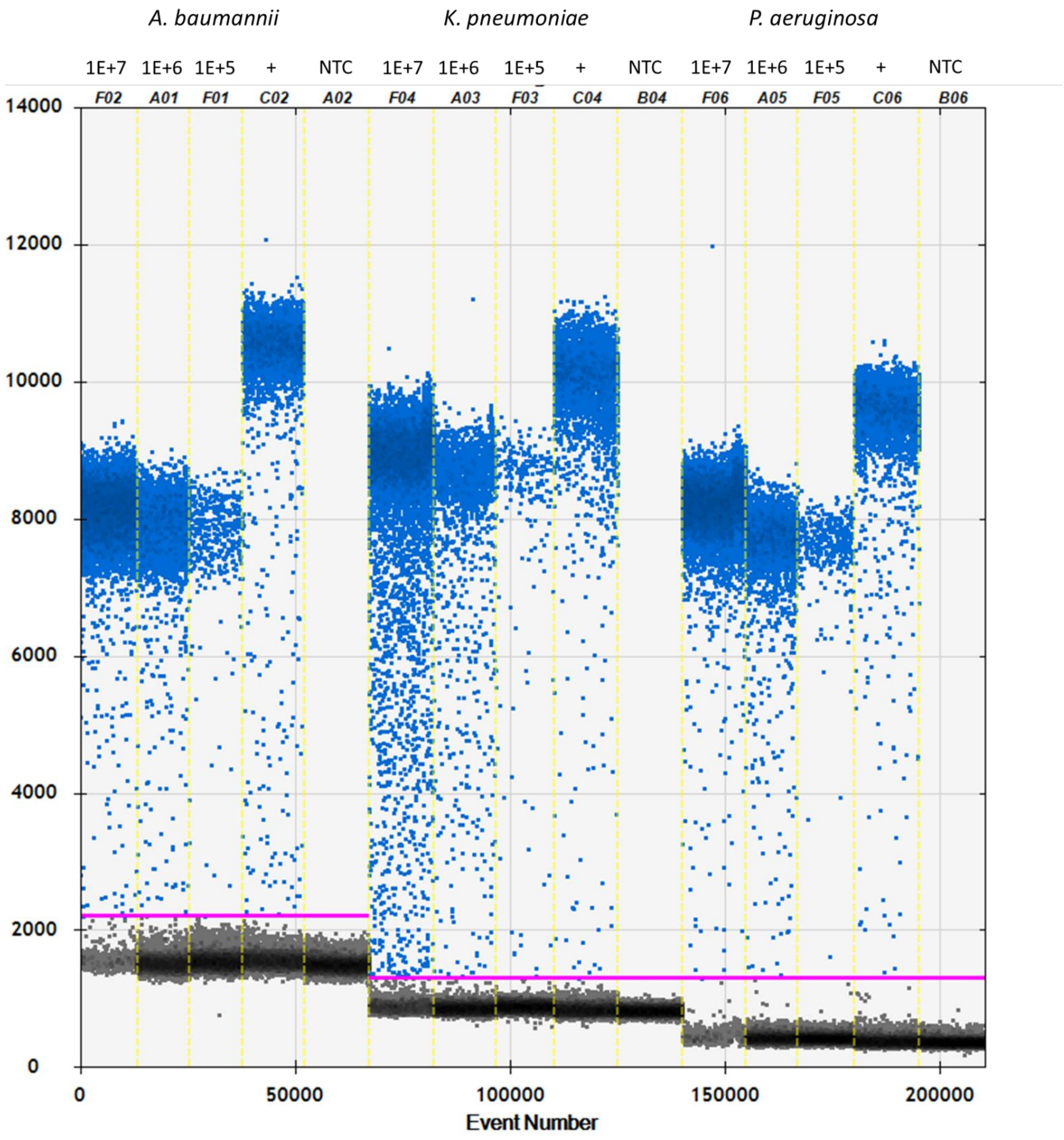

Figure S3: Direct dPCR for the three bacterial solutions used for the spiking of the sputum samples. 1E+7, 1E+6 and 1E+5 correspond to the concentrations in cells/mL based on turbidity measurements; + = positive control for corresponding target; NTC = non-template control; solid magenta line, threshold between positive and negative partitions (2200 for *A. baumannii*, 1300 for *K.pneumoniae* and *P. aeruginosa*)

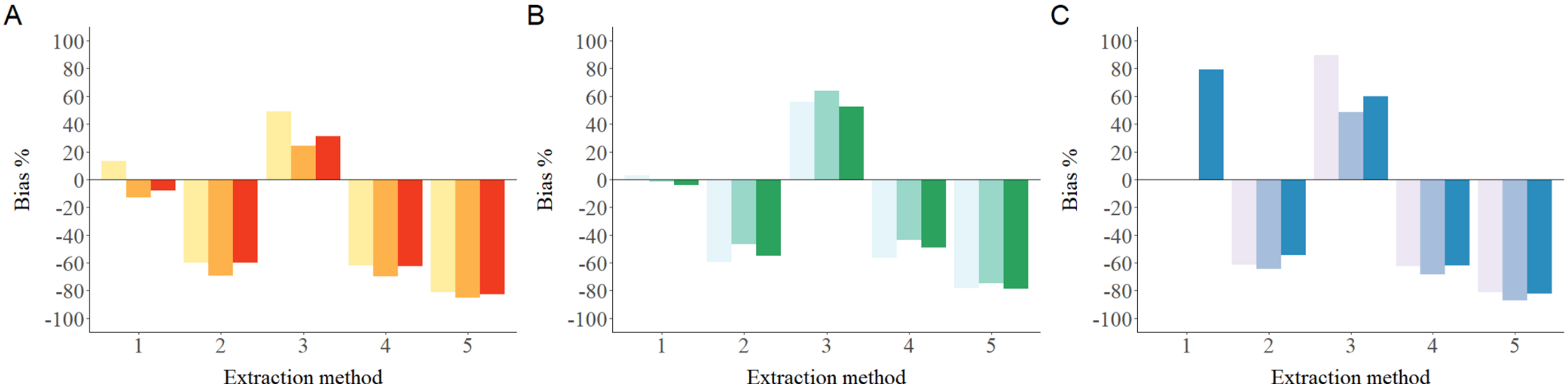

Figure S4: Bias to the theoretical 100% yields for the DNA extraction methods (1, CTAB; 2, QIAamp DNA mini kits; 3, GXT NA extraction kits; 4, QuickPick genomic DNA kits; 5, DNeasy UltraClean microbial kits) for the three bacteria (A, A. baumannii; B, K. pneumoniae; C, P. aeruginosa) and for the three samples (bacterial concentrations decrease from left to right). For the CTAB method for P. aeruginosa, the bias was too high to be depicted for high and middle bacterial concentrations.

Supplementary Tables

Table S1: Compositions of samples A, B and C (spiked sputum samples with added Liquillizer) based on the turbidity measurements and corrected using direct dPCR analysis.

**Sample**	**Bacterial concentration (cells/mL)**					
	A. baumannii		***K. pneumoniae***		***P. aeruginosa***	
	**Turbidity**	**Direct dPCR**	**Turbidity**	**Direct dPCR**	**Turbidity**	**Direct dPCR**
A	5.5E+04	1.3E+05	5.5E+05	5.4E+05	5.5E+03	1.2E+04
B	5.5E+05	1.2E+06	5.5E+03	5.7E+03	5.5E+04	1.3E+05
C	5.5E+03	1.3E+04	5.5E+04	5.3E+04	5.5E+05	1.2E+06

Table S2: Concentrations and coefficients of variation (CV) of the spiking solutions, as measured by direct dPCR.

**Estimated (cp/mL)**	**dPCR data**					
	A. baumannii		***K. pneumoniae***		***P. aeruginosa***	
	**(cp/mL)**	**CV (%)**	**(cp/mL)**	**CV (%)**	**(cp/mL)**	**CV (%)**
1E+05	2.42E+05	5.77	1.04E+05	4.90	2.16E+05	6.37
1E+06	2.43E+06	5.02	9.64E+05	3.46	2.27E+06	3.33
1E+07	2.10E+07	5.17	9.85E+06	9.59	2.20E+07	2.32

Table S3: Purity of the extracted DNA, based on spectrophotometric measurements.

Extraction method	Optical density ratio	
	**260/280**	**260/230**
CTAB	1.85	1.44
QIAamp DNA mini kits	1.87	1.94
GXT NA extraction kits	1.86	1.33
QuickPick genomic DNA kits	1.82	2.02
DNeasy UltraClean microbial kits	1.87	2.30

Table S4: Primers and probes used in the dPCR quantification.

Target[1]	Oligo	*Sequence*	*Amplicon length*	*Final concentration (nM)*
A. baumannii	F[2]	GCTCGTGATTCGACTCAAATCA		900
	R[3]	GCAAACGAATAATTTAACCATGCTT	68 bp	900
	P[4]	CTGATTAGCCAAGTTGC		300
K. pneumoniae	F	CCCGCTGTGGTAATACCCTACT		900
	R	GCTGCGTCAGGCAAATCTTC	62 bp	900
	P	CAGAGGGAACTGGC		300
P. aeruginosa	F	TCTGCCGCGGGTTCTTC		900
	R	AGGATACCTACGCCCAGTTGCT	64 bp	900
	P	CTTCCAGCAGGGACAC		300

[1]All assays were developed in-house, [2]Forward primer, [3]Reverse primer, [4]Probe – all probes were FAM/MGB

Table S5: Repeatability with coefficient of variance (CV) for *A. baumannii* for the three bacterial concentrations.

**Sample (100% theoretical yield)**	**Extraction method**	**Day 1**		**Day 2**		**Day 3**	
		**(cp/mL)**	**CV (%)**	**(cp/mL)**	**CV (%)**	**(cp/mL)**	**CV (%)**
A	CTAB	1.2E+05	8.4	1.1E+05	18.1	1.2E+05	6.8
(1.3E+05 cp/mL)	QIAamp DNA mini kits	3.3E+04	13.8	4.5E+04	15.5	4.5E+04	4.4
	GXT NA extraction kits	1.5E+05	7.1	1.8E+05	9.4	1.7E+05	5.0
	QuickPick genomic DNA kits	4.2E+04	8.2	4.3E+04	7.6	3.2E+04	19.8
	DNeasy UltraClean microbial kits	1.8E+04	13.8	1.9E+04	16.3	2.1E+04	4.1
B	CTAB	1.2E+06	6.2	1.0E+06	34.5	1.7E+06	33.8
(1.2E+06 cp/mL)	QIAamp DNA mini kits	3.9E+05	5.2	4.8E+05	9.9	5.1E+05	3.0
	GXT NA extraction kits	1.5E+06	2.3	1.9E+06	4.8	1.8E+06	4.3
	QuickPick genomic DNA kits	4.7E+05	18.8	5.1E+05	6.1	3.4E+05	25.1
	DNeasy UltraClean microbial kits	2.4E+05	6.7	2.0E+05	6.8	2.1E+05	19.9
C	CTAB	1.3E+04	29.8	1.3E+04	20.0	9.9E+03	26.9
(1.3E+04 cp/mL)	QIAamp DNA mini kits	5.1E+03	18.3	5.1E+03	31.8	5.8E+03	11.2
	GXT NA extraction kits	1.4E+04	11.7	2.0E+04	17.4	1.9E+04	12.6
	QuickPick genomic DNA kits	2.6E+03	25.6	2.1E+03	42.6	2.7E+03	44.1
	DNeasy UltraClean microbial kits	5.4E+03	11.2	5.4E+03	22.9	4.3E+03	12.9

Table S6: Repeatability with coefficient of variance (CV) for *K. pneumoniae* for the three bacterial concentrations.

Sample (100% theoretical yield)	Extraction method	Day 1		Day 2		Day 3	
		(cp/mL)	CV (%)	(cp/mL)	CV (%)	(cp/mL)	CV (%)
A (5.4E+05 cp/mL)	CTAB	5.5E+05	7.1	5.2E+05	17.2	6.0E+05	4.9
	QIAamp DNA mini kits	2.1E+05	4.1	2.2E+05	8.1	2.2E+05	4.4
	GXT NA extraction kits	6.9E+05	4.0	9.3E+05	3.3	9.1E+05	4.1
	QuickPick genomic DNA kits	2.2E+05	5.5	2.6E+05	11.0	2.2E+05	12.1
	DNeasy UltraClean microbial kits	1.1E+05	7.5	1.3E+05	10.4	1.2E+05	5.7
B (5.7E+03 cp/mL)	CTAB	5.9E+03	38.6	5.4E+03	36.9	5.2E+03	34.8
	QIAamp DNA mini kits	2.2E+03	40.9	2.3E+03	38.2	3.2E+03	40.4
	GXT NA extraction kits	7.5E+03	15.7	8.8E+03	14.6	9.8E+03	20.2
	QuickPick genomic DNA kits	2.7E+03	23.6	3.4E+03	24.7	2.6E+03	29.2
	DNeasy UltraClean microbial kits	1.4E+03	26.8	1.2E+03	32.9	1.1E+03	31.2
C (5.3E+04 cp/mL)	CTAB	4.8E+04	34.3	5.6E+04	13.6	5.2E+04	17.6
	QIAamp DNA mini kits	2.8E+04	13.3	2.6E+04	14.3	3.0E+04	13.7
	GXT NA extraction kits	6.8E+04	6.4	9.5E+04	7.6	9.7E+04	9.5
	QuickPick genomic DNA kits	1.4E+04	7.0	1.4E+04	25.4	1.1E+04	16.2
	DNeasy UltraClean microbial kits	2.9E+04	9.2	3.2E+04	18.5	2.9E+04	17.9

Table S7: Repeatability with coefficient of variance (CV) for *P. aeruginosa* for the three bacterial concentrations.

Sample (100% theoretical yield)	Extraction method	Day 1		Day 2		Day 3	
		(cp/mL)	CV (%)	(cp/mL)	CV (%)	(cp/mL)	CV (%)
A	CTAB	1.4E+05	64.4	1.4E+05	80.5	6.9E+05	25.0
(1.2E+03 cp/mL)	QIAamp DNA mini kits	4.7E+03	25.3	4.8E+03	15.6	4.2E+03	22.7
	GXT NA extraction kits	2.4E+04	43.5	2.1E+04	17.9	2.3E+04	15.4
	QuickPick genomic DNA kits	3.9E+03	20.0	5.6E+03	21.4	4.3E+03	29.9
	DNeasy UltraClean microbial kits	2.2E+03	26.0	2.3E+03	29.1	2.3E+03	17.1
B	CTAB	5.3E+05	40.2	4.5E+05	26.8	2.2E+06	22.9
(1.3E+04 cp/mL)	QIAamp DNA mini kits	4.1E+04	9.9	3.9E+04	14.3	5.4E+04	27.5
	GXT NA extraction kits	1.5E+05	4.2	2.0E+05	5.4	2.0E+05	6.6
	QuickPick genomic DNA kits	3.3E+04	19.0	4.0E+04	6.2	4.6E+04	48.9
	DNeasy UltraClean microbial kits	1.8E+04	13.8	1.7E+04	10.7	1.4E+04	22.4
C	CTAB	1.7E+06	17.9	1.8E+06	11.7	2.9E+06	42.0
(1.2E+05 cp/mL)	QIAamp DNA mini kits	5.5E+05	3.8	5.3E+05	5.8	5.5E+05	8.4
	GXT NA extraction kits	1.5E+06	3.3	2.1E+06	2.7	2.2E+06	8.1
	QuickPick genomic DNA kits	2.2E+05	9.0	2.2E+05	14.7	1.9E+05	9.3
	DNeasy UltraClean microbial kits	4.7E+05	14.1	4.8E+05	15.2	4.3E+05	8.9

Table S8: Intermediate precision with coefficient of variance (CV) for *A. baumannii* for the three bacterial concentrations.

Sample (100% theoretical yield)	Extraction method	Day 1 (cp/mL)	Day 2 (cp/mL)	Day 3 (cp/mL)	Mean (cp/mL)	CV (%)
A (1.3E+05 cp/mL)	CTAB	1.2E+05	1.1E+05	1.2E+05	1.2E+05	6.4
	QIAamp DNA mini kits	3.3E+04	4.5E+04	4.5E+04	4.1E+04	16.6
	GXT NA extraction kits	1.5E+05	1.8E+05	1.7E+05	1.7E+05	11.0
	QuickPick genomic DNA kits	4.2E+04	4.3E+04	3.2E+04	3.9E+04	15.7
	DNeasy UltraClean microbial kits	1.8E+04	1.9E+04	2.1E+04	2.0E+04	8.5
B (1.2E+06 cp/mL)	CTAB	1.2E+06	1.0E+06	1.7E+06	1.3E+06	28.1
	QIAamp DNA mini kits	3.9E+05	4.8E+05	5.1E+05	4.6E+05	13.2
	GXT NA extraction kits	1.5E+06	1.9E+06	1.8E+06	1.7E+06	12.7
	QuickPick genomic DNA kits	4.7E+05	5.1E+05	3.4E+05	4.4E+05	19.5
	DNeasy UltraClean microbial kits	2.4E+05	2.0E+05	2.1E+05	2.2E+05	12.1
C (1.3E+04 cp/mL)	CTAB	1.3E+04	1.3E+04	9.9E+03	1.2E+04	16.3
	QIAamp DNA mini kits	5.1E+03	5.1E+03	5.8E+03	5.3E+03	7.8
	GXT NA extraction kits	1.4E+04	2.0E+04	1.9E+04	1.7E+04	17.0
	QuickPick genomic DNA kits	5.4E+03	5.4E+03	4.3E+03	5.0E+03	13.1
	DNeasy UltraClean microbial kits	2.6E+03	2.1E+03	2.7E+03	2.5E+03	12.4

Table S9: Intermediate precision with coefficient of variance (CV) for *K. pneumoniae* for the three bacterial concentrations.

**Sample (100% theoretical yield)**	**Extraction method**	**Day 1 (cp/mL)**	**Day 2 (cp/mL)**	**Day 3 (cp/mL)**	**Mean (cp/mL)**	**CV (%)**
A	CTAB	5.5E+05	5.2E+05	6.0E+05	5.6E+05	7.8
(5.4E+05 cp/mL)	QIAamp DNA mini kits	2.1E+05	2.2E+05	2.2E+05	2.2E+05	2.6
	GXT NA extraction kits	6.9E+05	9.3E+05	9.1E+05	8.5E+05	16.0
	QuickPick genomic DNA kits	2.2E+05	2.6E+05	2.2E+05	2.4E+05	10.1
	DNeasy UltraClean microbial kits	1.1E+05	1.3E+05	1.2E+05	1.2E+05	11.0
B	CTAB	5.9E+03	5.4E+03	5.2E+03	5.5E+03	6.3
(5.7E+03 cp/mL)	QIAamp DNA mini kits	2.2E+03	2.3E+03	3.2E+03	2.6E+03	19.9
	GXT NA extraction kits	7.5E+03	8.8E+03	9.8E+03	8.7E+03	13.3
	QuickPick genomic DNA kits	2.7E+03	3.4E+03	2.6E+03	2.9E+03	14.0
	DNeasy UltraClean microbial kits	1.4E+03	1.2E+03	1.1E+03	1.2E+03	11.7
C	CTAB	4.8E+04	5.6E+04	5.2E+04	5.2E+04	7.8
(5.3E+04 cp/mL)	QIAamp DNA mini kits	2.8E+04	2.6E+04	3.0E+04	2.8E+04	7.9
	GXT NA extraction kits	6.8E+04	9.5E+04	9.7E+04	8.7E+04	18.8
	QuickPick genomic DNA kits	2.9E+04	3.2E+04	2.9E+04	3.0E+04	4.6
	DNeasy UltraClean microbial kits	1.4E+04	1.4E+04	1.1E+04	1.3E+04	13.8

Table S10: Intermediate precision with coefficient of variance (CV) for *P. aeruginosa* for the three bacterial concentrations.

**Sample (100% theoretical yield)**	**Extraction method**	**Day 1 (cp/mL)**	**Day 2 (cp/mL)**	**Day 3 (cp/mL)**	**Mean (cp/mL)**	**CV (%)**
A	CTAB	1.4E+05	1.4E+05	6.9E+05	3.2E+05	96.9
(1.2E+04 cp/mL)	QIAamp DNA mini kits	4.7E+03	4.8E+03	4.2E+03	4.6E+03	7.2
	GXT NA extraction kits	2.4E+04	2.1E+04	2.3E+04	2.3E+04	7.5
	QuickPick genomic DNA kits	3.9E+03	5.6E+03	4.3E+03	4.6E+03	20.3
	DNeasy UltraClean microbial kits	2.2E+03	2.3E+03	2.3E+03	2.2E+03	3.2
B	CTAB	5.3E+05	4.5E+05	2.2E+06	1.1E+06	94.6
(1.3E+05 cp/mL)	QIAamp DNA mini kits	4.1E+04	3.9E+04	5.4E+04	4.5E+04	17.8
	GXT NA extraction kits	1.5E+05	2.0E+05	2.0E+05	1.9E+05	14.9
	QuickPick genomic DNA kits	3.3E+04	4.0E+04	4.6E+04	4.0E+04	17.2
	DNeasy UltraClean microbial kits	1.8E+04	1.7E+04	1.4E+04	1.6E+04	10.6
C	CTAB	1.7E+06	1.8E+06	2.9E+06	2.1E+06	29.7
(1.2E+06 cp/mL)	QIAamp DNA mini kits	5.5E+05	5.3E+05	5.5E+05	5.4E+05	2.5
	GXT NA extraction kits	1.5E+06	2.1E+06	2.2E+06	1.9E+06	18.4
	QuickPick genomic DNA kits	4.7E+05	4.8E+05	4.3E+05	4.6E+05	6.0
	DNeasy UltraClean microbial kits	2.2E+05	2.2E+05	1.9E+05	2.1E+05	6.9

Minimum Information for Publication of Quantitative Digital PCR Experiments for 2020" (dMIQE2020) checklist

ITEM TO CHECK	PROVIDED	COMMENT
**1. SPECIMEN**		
Detailed description of specimen type and numbers	Y	Materials and methods
Sampling procedure (including time to storage)	N	
Sample aliquoting, storage conditions and duration	Y	Materials and methods
**2. NUCLEIC ACID EXTRACTION**		
Description of extraction method including amount of sample processed	Y	Materials and methods
Volume of solvent used to elute/resuspend extract	Y	Materials and methods
Number of extraction replicates	Y	Materials and methods
Extraction blanks included?	Y	Materials and methods
**3. NUCLEIC ACID ASSESSMENT AND STORAGE**		
Method to evaluate quality of nucleic acids	Y	Materials and methods
Method to evaluate quantity of nucleic acids (including molecular weight and calculations when using mass)	Y	Materials and methods
Storage conditions: temperature, concentration, duration, buffer, aliquots	Y	Materials and methods
Clear description of dilution steps used to prepare working DNA solution	Y	Materials and methods
**4. NUCLEIC ACID MODIFICATION**	**NA**	
Template modification (digestion, sonication, pre-amplification, bisulphite, etc.)		
Details of repurification following modification, if performed		
**5. REVERSE TRANSCRIPTION**	**NA**	
cDNA priming method and concentration		
One or two step protocol (include reaction details for two step)		
Amount of RNA added per reaction		
Detailed reaction components and conditions		
Estimated copies measured with and without addition of reverse transcriptase		
Manufacturer of reagents used, with catalogue and lot numbers		
Storage of cDNA: temperature, concentration, duration, buffer and aliquots		
**6. dPCR OLIGONUCLEOTIDES DESIGN AND TARGET INFORMATION**		
Sequence accession number or official gene symbol	N	
Method (software) used for design and *in-silico* verification	N	
Location of amplicon	N	
Amplicon length	Y	Supplementary Information
Primer and probe sequences (or amplicon context sequence)	Y	Supplementary Information
Location and identity of any modifications	Y	Supplementary Information
Manufacturer of oligonucleotides	Y	Oligos were from Integrated DNA Technologies
**7. dPCR PROTOCOL**		

Manufacturer of dPCR instrument and instrument model	Y	Materials and methods
Buffer/kit manufacturer with catalogue and lot number	Y	Materials and methods
Primer and probe concentrations	Y	Materials and methods
Pre-reaction volume and composition (including amount of template and if restriction enzyme added)	Y	Materials and methods
Template treatment (initial heating or chemical denaturation)	N	No template treatment prior to PCR
Polymerase identity and concentration, Mg^{++} and dNTP concentrations	N	Not disclosed by manufacturer
Complete thermocycling parameters	Y	Materials and methods
**8. ASSAY VALIDATION**	**NA**	
Details of optimisation performed		
Analytical specificity (vs. related sequences) and limit of blank		
Analytical sensitivity/limit of detection, and how this was evaluated		
Testing for inhibitors (from biological matrix/extraction)		
**9. DATA ANALYSIS**		
Description of dPCR experimental design	Y	Materials and methods
Comprehensive details of negative and positive controls (whether applied for quality control or for estimation of error)	Y	Materials and methods
Partition classification method (thresholding)	N	
Examples of positive and negative experimental results (including fluorescence plots in Supplemental Materials)	Y	Supplementary Figures
Description of technical replication	Y	Materials and methods
Repeatability (intra-experiment variation)	Y	Materials and methods
Reproducibility (inter-experiment/user/lab, etc. variation)	Y	Materials and methods
Number of partitions measured (average and standard deviation)	N	14311 (CV = 9.08 %)
Partition volume	Y	0.715 nL, as measured by Bogožalec Košir et al., 2017
Copies per partition (λ or equivalent) (average and standard deviation)	Y	Large variability due to a wide concentration range tested. *A. baumannii* – 0.08705 (min = 0.00032, max = 0.56943) *K. pneumoniae* – 0.04413 (min = 0.00013, max=0.28274) *P. aeruginosa* – 0.11361 (min = 0.00067, max = 0.69224)
dPCR analysis program (source, version)	Y	Materials and methods
Description of normalisation method	N	No normalisation used
Statistical methods used for analysis	Y	Materials and methods
Data transparency	Y	
